# Kikuchi-Fujimoto disease associated with a flare-up of a leukocytoclastic vasculitis: A rare case report and review of literature

**DOI:** 10.1097/MD.0000000000037626

**Published:** 2024-03-29

**Authors:** Nicolas Sandakly, Georgio El Koubayati, Jeannette Sarkis, Samah Naderi, Delivrance Sebaaly, Fady Haddad

**Affiliations:** a Faculty of Medical Sciences, Lebanese University, Hadath Campus, Hadath, Lebanon; b Department of Internal Medicine, Lebanese Hospital Geitaoui University Medical Center, Beirut, Lebanon; c Department of Pathology, Lebanese Hospital Geitaoui University Medical Center, Beirut, Lebanon.

**Keywords:** Kikuchi-Fujimoto, leukocytoclastic vasculitis, lymphadenopathy

## Abstract

**Rationale:**

Kikuchi-Fujimoto disease (KFD) also known as histiocytic necrotizing lymphadenopathy is an exceedingly rare cause of cervical lymphadenopathy, commonly accompanied by systemic symptoms such as fever, fatigue, night sweats, myalgia, skin rash.

**Patient concerns:**

In this paper, we report the case of a 22-year-old female patient who experienced a flare-up of leukocytoclastic vasculitis that was complicated by the appearance of a cervical lymph node with dysphagia, fever and nausea.

**Diagnosis:**

Infectious and autoimmune workup came back negative.

**Interventions:**

Excisional lymph node biopsy was done and the pathology results were consistent with histiocytic necrotizing lymphadenitis in keeping with Kikuchi-Fujimoto disease.

**Outcomes:**

Patient improved on intravenous corticosteroids and was discharged on per os prednisone. Six month follow-up shows complete resolution of her symptoms.

**Lessons:**

KFD should be ruled out in patients with autoimmune or inflammatory diseases who develop lymphadenopathies.

## 1. Introduction

Lymphadenopathy is not a disease per se, rather it denotes the enlargement of the lymph nodes in response to an underlying etiology. Cervical lymphadenopathies are prevalent, given that 40% of the body’s total lymph nodes are located in the head and neck.^[1]^ Etiologies comprise malignancy, infectious (including viral, bacterial, and fungal pathogens), and autoimmune disorders, as well as medications and iatrogenic causes. Although history and physical examination alone can assist in identifying the underlying cause, further investigation including a lymph node biopsy is warranted when the underlying cause remains unknown. Kikuchi-Fujimoto disease (KFD) also known as histiocytic necrotizing lymphadenopathy is an exceedingly rare cause of cervical lymphadenopathy. Patients may also present with symptoms such as fever, fatigue, night sweats, myalgia, skin rash. It is a benign self-limited disease that is clinically confirmed by histopathology and differentiated from other infectious and noninfectious conditions.^[2]^ According to Dorfman et al,^[3]^ 30% of patients with KFD were initially misdiagnosed as lymphoma. Infrequently observed, the association of KFD with autoimmune diseases, in particular, such as systemic lupus erythematosus. We herein describe the case of a 22-year-old female patient who experienced a flare-up of leukocytoclastic vasculitis and was subsequently diagnosed with Kikuchi disease

## 2. Case presentation

**Figure 1.**
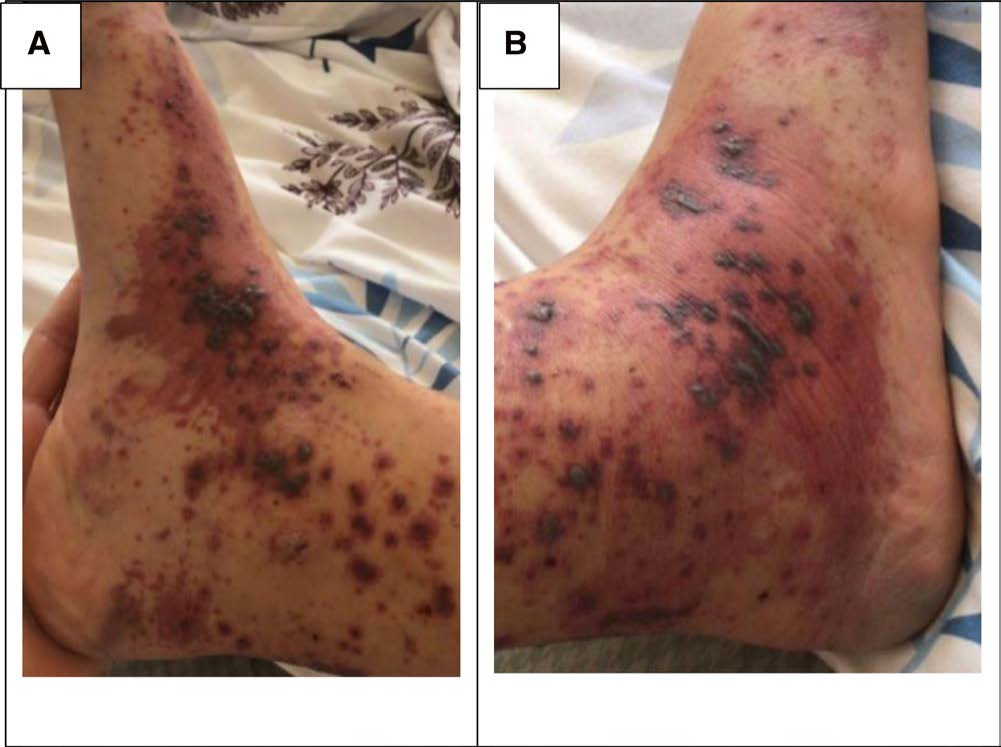
Medial (A) and lateral (B) aspect of the feet and ankles showing purpuric hemorrhagic and bullous lesions indicating flare up of the previously diagnosed leukocytoclastic vasculitis.

A 22-year-old female patient was admitted to the Lebanese Hospital Geitaoui University Medical Center (LHG-UMC) for a 7-day history of bilateral palpable purpura of the lower extremities preceded by 5 days of arthralgia and sore throat. Her medical history was significant for leukocytoclastic vasculitis in remission for the past 2 years. Upon admission, vital signs were within reference range. Physical examination was relevant for purpuric lesions on the lower limbs, which were painful and pruritic. The lesion was hemorrhagic and bullous over the medial and lateral aspects of both ankles (Fig. 1).

**Figure 2.**
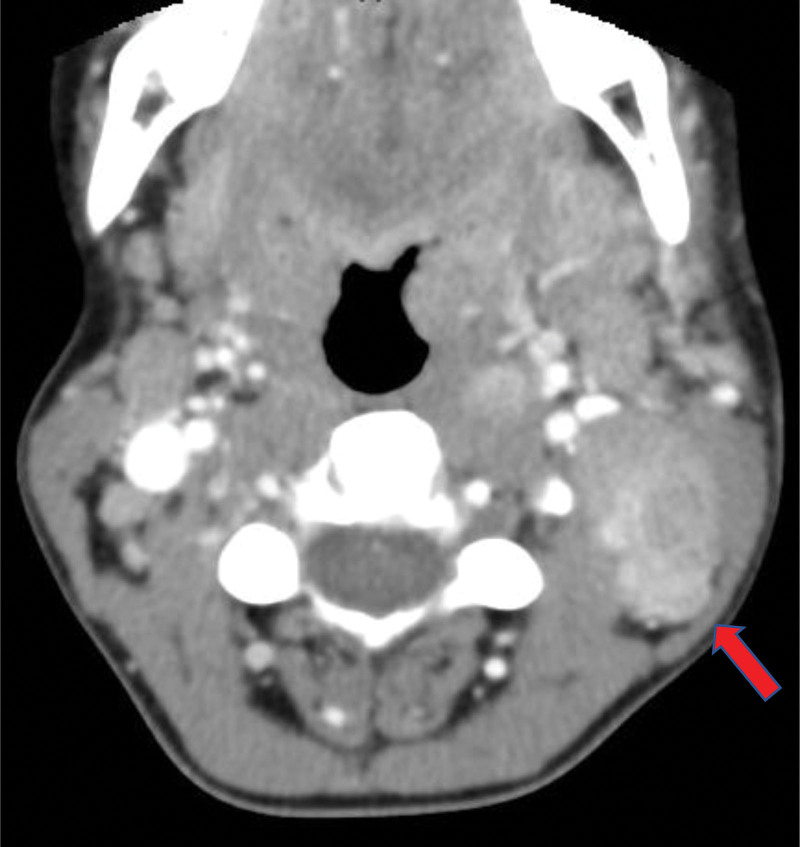
Contrast enhanced cervical CT scan showing enlarged cervical lymph nodes (red arrow) in the left para carotid space, compressing on the posterior wall of the left internal jugular vein, showing heterogenous parenchyma.

Initial laboratory studies indicated a hemoglobin level of 10.5 g/dL (reference range of 12–16 g/dL) and leukocytosis at 11,210 cells/mm^3^ (4800–10,800 cells/mm^3^). Liver and renal function tests were within the reference range. The erythrocyte sedimentation rate was prolonged at 24 mm/1st hour. The patient was started on clobetasol propionate cream twice daily, along with intravenous (IV) methylprednisolone 40 mg once daily and ceftriaxone 2 g IV daily and clindamycin 300 mg IV every 8 hours for 5 days. Over the subsequent 4 days, her symptoms gradually improved. However, on the fifth day of hospitalization, she noticed a lump in the left cervical region and reported nausea and dysphagia. The patient was febrile at 38.8 °C. Physical examination revealed bilateral erythematous, non-exudative tonsils, more prominent on the left side. A left upper cervical lymph node that is mobile, tender, and firm was also noted. She denied any history of travel or animal contact. A contrast-enhanced computer tomography scan of the neck (Fig. 2) revealed enlarged cervical lymph nodes in the left para carotid space, compressing on the posterior wall of the left internal jugular vein, showing heterogenous parenchyma.

**Figure 3.**
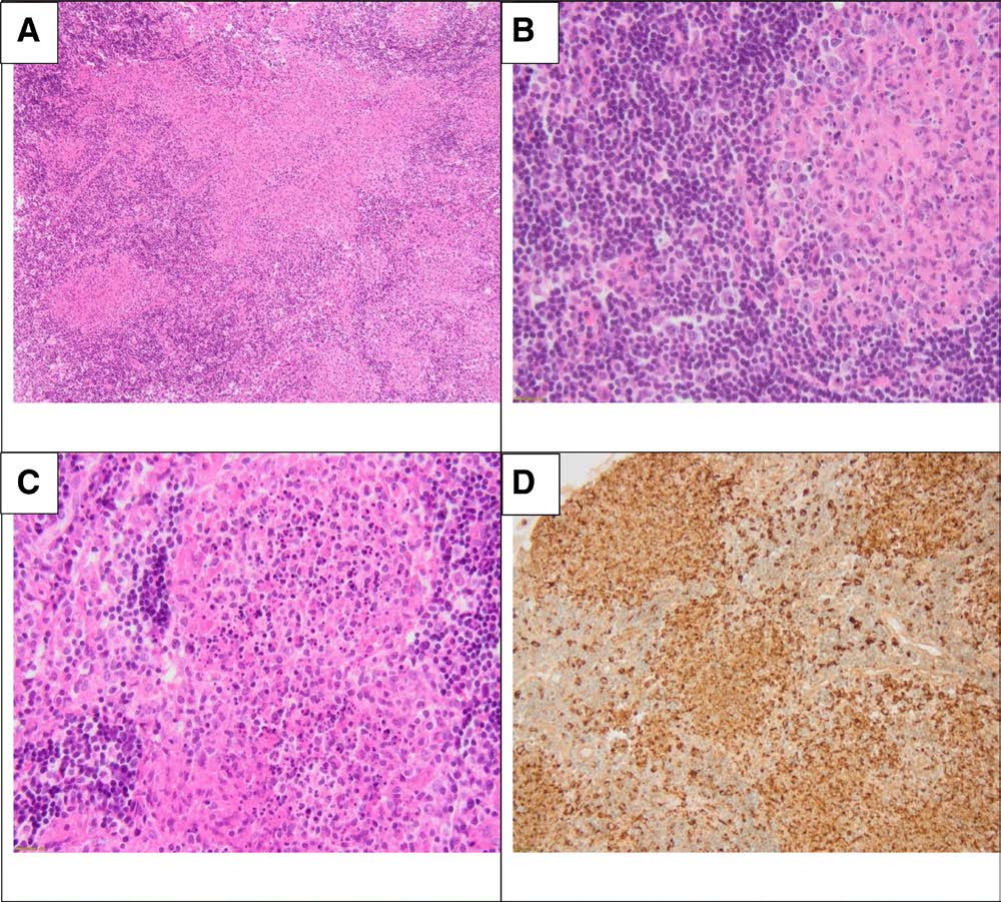
Histopathological findings of the left cervical lymph node. (A) The lymph node shows partial effacement by irregularly shaped pale staining areas(hematoxylin and eosin stain, 100×). (B) In the right upper of the image: pale staining area composed of histiocytes, and abundant karyorrhectic debris. To the left: hyperplasia of the post-capillary venules, and numerous immunoblasts. (hematoxylin and eosin stain, 400×). (C) High power field showing an area of epithelioid histiocytes with a central collection of numerous neutrophils (hematoxylin and eosin stain, 400×). (D) Intense and diffuse expression of CD68 stain (200×).

Blood tests revealed an elevated C-reactive protein (CRP) at 51 mg/L (reference range < 6 mg/L), and a negative procalcitonin level. Erythrocyte sedimentation rate was further prolonged at 62 mm/1st hour. The patient underwent an excisional lymph node biopsy. Serological tests for cytomegalovirus (CMV) and Epstein-Barr virus returned negative findings, as were for streptococcus group A. Autoimmune work-up came back negative (Table 1). Blood and sputum cultures retrieved during the febrile episode yielded negative results. Histopathology findings were consistent with histiocytic necrotizing lymphadenitis in keeping with Kikuchi-Fujimoto disease (Fig. 3). The patient was continued on IV methylprednisolone and was discharged 1 week later on a 6 week taper of per os Prednisone 30mg, without any adverse effects reported.

**Table 1:** Auto-immune work-up.

Investigation	Results	Normal values
Serum immunoglobulin (Ig)		
IgA (g/L)	12.95	0.8–3
IgM (g/L)	1.01	0.4–2.5
IgG (g/L)	15.78	6–16
ANA	1/100	<1/160
SS-A native (SSA) (µ/mL)	14.6	0–91
SS-B (SSB) (µ /mL)	13.1	0–73
Scl-70 (Scl) (AU/mL)	3.4	<29
Jo-1 (Jo) (AU/mL)	2	<20
Nucleosomes (NUC)	Negative	Not available
Histones (HI) (U/mL)	2	<40
Anti-ds-DNA IgG (IU/mL)	10.9	0–29.9
Anti Sm (U/mL)	3.2	0–7
ANCA-C (AU/mL)	2.8	≤19
ANCA-P (kU/L)	1.2	<1.4
C3 (g/L)	1.2	0.9–1.8
C4 (g/L)	0.12	0.1–0.4
Anti-B2-glycoprotein IgM (SMU/mL)	4.8	≤20
Anti-cardiolipin IgG (GPL U/mL)	27.5	≤15
Anti-cardiolipin IgM (MPL U/mL)	1.1	<12.5
Anti-cardiolipin IgA (APL U/mL)	2	≤11
Antiphospholipid IgG (U/mL)	13.4	<14
Antiphospholipid IgM (U/mL)	1.2	<14

**Figure 4.**
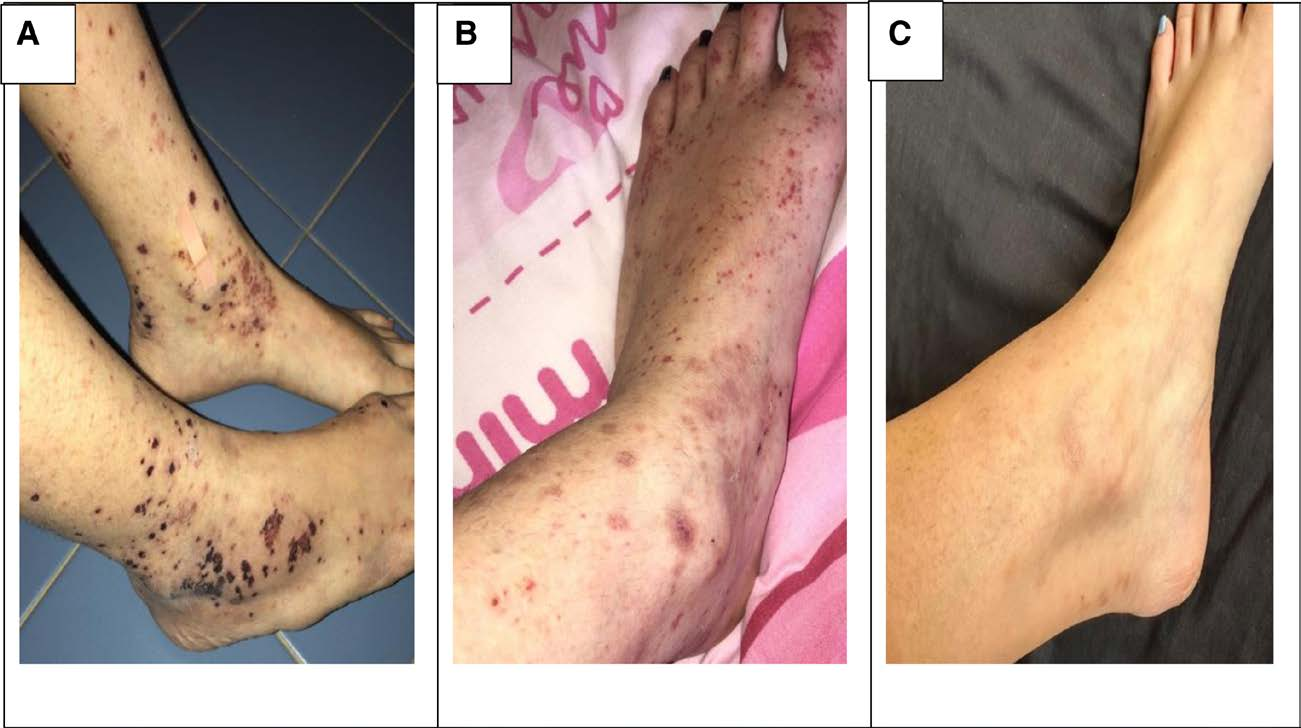
Evolution of the purpuric lesions over 1 month (A), 2 months (B) and 6 months (C) post discharge from the hospital showing complete resolution of the lesions.

At 6-month follow-up, the patient was doing well, with complete resolution of her lymphadenitis and rash, and there were no manifestations of connective tissue diseases (Fig. 4).

## 3. Discussion

Kikuchi-Fujimoto disease (KFD) has been documented worldwide in the literature, since its first description in Japan in 1972 by the pathologists Kikuchi and Fujimoto. A retrospective study including 330 reported cases of KFD retrieved from PubMed over a 14-year period, revealed that approximately 50% of these cases originated from East Asia, with a mere 7% reported in America.^[2]^ Another retrospective study focusing on KFD and its prevalence in the Eastern Mediterranean regions, indicated a lower rate compared to the Asian communities with around 60% of the reported cases originating from the Arabian Gulf states.^[4]^ Cervical lymphadenopathy is the most commonly presenting symptom of KFD, commonly associated with fever. Although quite rare, axillary, supraclavicular,^[5]^ mesenteric,^[6]^ and mediastinal^[7]^ lymphadenopathy, have been described as a presenting manifestation of KFD. The diagnosis is confirmed through histopathology. Lymph nodes exhibit features of follicular hyperplasia with partially preserved architecture. Histological lesions comprise necrosis of the paracortical zone with the presence of numerous histiocytes, including plasma cells and large lymphoid cells, along with abundant karyorrhectic nuclear debris but no polymorphonuclear neutrophils and no caseous necrosis. Immunohistochemical analysis demonstrates that the cells present in pathological zones of the node are mainly CD3+ cells and CD68+ histiocytes. The cell population is predominantly composed of CD68+ histiocytes, while the lymphocyte population represents 20% to 50% of the total cell population, mainly CD8+ cytotoxic T lymphocytes.^[8]^ These histological features were present in our patients, with high CD68 expression reflecting histiocyte proliferation. The exact etiology of histiocytic necrotizing lymphadenitis remains unclear. Various infectious agents have been hypothesized to be involved in the pathogenesis of the disease. The benign self-limited course of the disease, coupled with its poor response to antibiotics, supported this hypothesis. However, there is no conclusive evidence in the literature validating a causal relationship between a specific agent and KFD. A recent meta-analysis aimed at evaluating the association of infectious agents with KFD found no association to Epstein-Barr virus positivity.^[9]^ However, while parvovirus B19 and human herpes virus 8 (HHH-8) positivity seems to be related to KFD, the limited number of studies poses a limitation in drawing a definitive conclusion.^[9]^ Notably, several authors have reported KFD following either COVID-19 infection or covid-19 vaccines.^[10,11]^ Another hypothesized theory is its link with autoimmune diseases, mainly systemic lupus erythematosus (SLE). In a systematic literature review including 113 adults with KFD and SLE diagnosis, retrieved from 80 studies, Bernard et al^[12]^ identified 3 patterns of association: SLE was diagnosed before KFD in 18% of the cases, concurrent diagnosis was made in 51%, and in 31% of the cases, SLE was diagnosed after KFD diagnosis. In this case, we describe a 22-year-old female patient who presented for a flare-up of her small vessel vasculitis and was found to have KFD on histopathological studies of cervical lymph node biopsy. Leukocytoclastic vasculitis is thought to be related to viral and bacterial etiologies,^[13]^ which have also been described in association with histiocytic necrotizing lymphadenitis. Moreover, our patient had upper respiratory tract manifestations which could have been the trigger of her both conditions and the activation of a systemic immune inflammatory reaction. This association is extremely rare among patients with histiocytic necrotizing lymphadenitis. A thorough systematic search on PubMed/Medline using the MeSH term Kikuchi disease, Kikuchi-Fujimoto disease, histiocytic necrotizing lymphadenitis, and leukocytoclastic vasculitis, small vessel vasculitis yielded just 4 documented cases to which we added our case (Table 2). Kawai et al^[14]^ reported a 10-year-old girl who was diagnosed with KFD and during her hospital stay, petechiae on the soles of her feet were observed and found to have leukocytoclastic vasculitis on skin biopsy. In a cohort of 20 patients diagnosed with KFD, Sopeña et al^[15]^ reported 2 patients who were found to have small vessel vasculitis; the first patient’s diagnosis was incidentally observed in the perinodal fat tissue, and the second patient during her first episode of KD presented with skin lesions in the preauricular region and cheeks and biopsy showed leukocytoclastic vasculitis. Four months later, she developed SLE coincident with the first recurrence of KFD. Famularo et al^[16]^ described a 27-year-old woman with two-biopsy proven episodes of KFD occurring 16 years apart, and on the second occasion, lymph node biopsy showed leukocytoclastic vasculitis in addition to the typical features of KFD. Although it is a rare disease, it should be considered in the differential diagnosis for patients with fever and lymphadenopathy, especially in cases of febrile cervical lymphadenopathy where improvement is not observed with antibiotics. While KFD is a benign self-limited disease, it is important to bear in mind the association with vasculitis and other connective tissue disease during follow-ups.

**Table 2:** Summary of clinical manifestations of reported KFD associated with leukocytoclastic vasculitis.

			Kikuchi-Fujimoto disease					Leukocytoclastic vasculitis	
Case	Gender	Age (Year)	Symptoms durations	Autoimmunity work-up	Treatment	Clinical outcome	Follow-up	Latency of the diagnosis	Confirmed diagnosis
Kawai et al^[14]^	Female	10	One month	ANA: Positive (1/640) Anti-DNA Antibodies: negative	Prednisolone 2 mg/kg per day for 2 weeks	Improvement	No evidence of relapse or auto-immune disease after 2-year of follow-up ANA: negative	After the diagnosis of KFD	Biopsy of the skin lesion over the sole
Sopeña et al^[15]^	Male	Not available	Not available	Not available	Not available	Complete recovery	No evidence of relapse or auto-immune diseases after 118 months of follow-up	Simultaneous diagnosis of KFD and leukocytoclastic vasculitis	Biopsy of the inguinal lymph node
Sopeña et al^[15]^	Female	Not available	Not available	Not available	Not available	Complete recovery	Recurrence of KFD coincident with SLE diagnosis at 6-month follow-up	Simultaneous diagnosis of leukocytoclastic vasculitis and the first episode of KFD	Biopsy of the skin lesion in the periauricular region and cheeks
Famularo et al^[16]^	Female	27	7 weeks	ANA, RF, ANCA, antiphospholipid antibodies: negative	Tapering prednisone 5 mg/kg per day for 13 weeks	Complete recovery	No evidence of relapse or auto-immune diseases after 6-month of follow-up while still on prednisone (25 mg daily)	Simultaneous diagnosis of KFD and leukocytoclastic vasculitis	Biopsy of the lymph node

## 4. Take away lessons

KFD is an extremely rare cause of lymphadenopathy associated with fever, myalgia and rash. Its association with autoimmune and inflammatory and vasculitic diseases is well reported. It is usually benign and self-limited.

## Author contributions

**Conceptualization:** Nicolas Sandakly, Jeannette Sarkis.

**Data curation:** Samah Naderi, Delivrance Sebaaly.

**Investigation:** Nicolas Sandakly, Georgio El Koubayati.

**Methodology:** Nicolas Sandakly, Georgio El Koubayati, Jeannette Sarkis.

**Resources:** Delivrance Sebaaly.

**Supervision:** Fady G. Haddad.

**Validation:** Georgio El Koubayati, Fady G. Haddad.

**Visualization:** Nicolas Sandakly, Georgio El Koubayati, Samah Naderi, Fady G. Haddad.

**Writing – original draft:** Nicolas Sandakly, Jeannette Sarkis, Fady G. Haddad.

**Writing – review & editing:** Georgio El Koubayati, Fady G. Haddad.
